# Africa and climate justice at COP27 and beyond: impacts and solutions through an interdisciplinary lens

**DOI:** 10.14324/111.444/ucloe.000062

**Published:** 2023-08-18

**Authors:** Jhénelle Williams, Simon Chin-Yee, Mark Maslin, Jonathan Barnsley, Anthony Costello, John Lang, Jacqueline McGlade, Yacob Mulugetta, Richard Taylor, Matthew Winning, Priti Parikh

**Affiliations:** 1 Department of Geography, North-West Wing, University College London, Gower Street, London WC1E 6BT, UK; 2 Department of Political Science, The School of Public Policy, University College London, The Rubin Building, 29/31 Tavistock Square, London WC1H 9QU, UK; 3 Institute for Global Health, Institute of Child Health, University College London, 30 Guilford Street, London WC1N 1EH, UK; 4 Energy and Climate Intelligence Unit, 180 Borough High St, London SE1 1LB, UK; 5 Institute for Global Prosperity, University College London, Maple House, 149 Tottenham Court Road, London W1T7NF, UK; 6 Department of Science, Technology, Engineering and Public Policy (STEaPP), University College London, UK; 7 UCL Institute for Sustainable Resources, University College London, 14 Upper Woburn Place, London WC1H 0NN, UK; 8 Engineering for International Development Centre, Bartlett School of Sustainable Construction, University College London, 1–19 Torrington Place, London WC1E 7HB, UK

**Keywords:** climate justice, climate change, Africa, sustainable development, climate finance, gender, environment, policy, energy, health

## Abstract

Climate justice is not just a financial transaction to protect the environment. It needs to be seen as the protection of the most vulnerable in society after centuries of resource exploitation. African countries disproportionately face impacts of climate change on their environments, their economies, their resources and their infrastructure. This leads to greater vulnerability and increased exposure to the negative effects of a changing climate. In this article, we highlight the importance of climate justice and its role within the United Nations negotiations, and ultimately in concrete action. We discuss current climate impacts across key sectors in the African region, with a focus on health, infrastructure, food and water scarcity, energy and finance. All sectors are affected by climate change. They are interconnected and under threat. This triggers a ripple effect, where threats in one sector have a knock-on effect on other sectors. We find that the current set of intergovernmental institutions have failed to adequately address climate justice. We also contend that a siloed approach to climate action has proven to be ineffective. As we head towards the next set of negotiations (COP27), this paper argues that the economic and social conditions in Africa can be addressed through financial and collaborative support for adaptation and localised solutions, but that this will only be achieved if climate justice is prioritised by the decision makers. This needs to include a global-scale transition in how climate finance is assessed and accessed. Climate justice underpins real, effective and sustainable solutions for climate action in Africa.

## Introduction

Global warming has triggered changes in the Earth’s climate including an increasing frequency and intensity of extreme weather events such as heatwaves, droughts, floods and tropical cyclones. In 2022, average global temperatures sit at ~1.1°C above the pre-industrial average and, according to the most recent Intergovernmental Panel on Climate Change (IPCC) report, we are likely to breach the 1.5°C global target within the next decade. These increasing temperatures contribute to rising cases of heat stress with heatwaves worsening annually. According to the most recent *Lancet* report, vulnerable populations faced almost 4 billion more person-days (*refers to the number of days per year that exceed a heat exposure threshold multiplied by the total population that was exposed* [1,2]) of heatwave exposure in 2021 as compared to the 1986–2005 period. Hence, wildfires are increasing in most regions; and heat affects the productivity, health and livelihoods of agricultural and construction workers [2]. Furthermore, heat stress and changing rainfall patterns have added to the impact on food prices arising from conflict around the world. With Africa having a large proportion of its population living in drylands [3], the current and projected increase in dryland areas (especially those at risk for desertification) has a deteriorating effect on child hunger, increased population migration and negative impacts on agriculture and livestock [2]. Scientific assessments on the drivers and impacts of climate change published by the IPCC, the Intergovernmental Science-Policy Platform on Biodiversity and Ecosystem Services (IPBES) [4] and numerous other intergovernmental bodies have highlighted not only the urgency of taking action to curb emissions, but also that future policy developments and interventions ensure more equitable and sustainable outcomes [5].

While the Glasgow Climate Pact, adopted by member states at COP26, promotes a more ambitious response to the climate crisis, such as making additional funding available for adaptation, an agreement to phasedown unabated coal power and the phase out of inefficient fossil fuels subsidies, as well as support for loss and damage, there was no consensus on how to reverse or avoid the catastrophic impacts of anthropogenic climate change. Without adequate financing, human and ecosystem health, food security, water scarcity and infrastructure are all at risk, especially in least developed countries (LDCs) [6,7]. Human health is a particular concern for those who live in informal housing as well as those forced to take up informal employment to survive [8]. There is a considerable failure in the Glasgow Climate Pact to highlight the need for tailored financial and social frameworks that will enable and accelerate adaptation in a fair and just manner.

COP27 Sharm El Sheikh is being framed as an African COP, putting in full view the consequences of climate change across Africa. The continued sidelining of voices from LDCs and those most vulnerable in society is detrimental not only to those communities and regions but also to the solidarity of global climate action. This paper adds to those voices advocating for a transition in climate action to one where justice is at the core. Recognising the gross inequity between the impacts of, and responsibility for, climate change, we aim to bring attention to the critical need for climate justice to be at the heart of all deliberations for the continent whether it is COP27 or future negotiations.

## Framing climate justice

As climate justice is the central thread of this paper, we begin by outlining the genesis and foundation of what climate justice means. Climate justice was born out of the wider environmental justice movements that saw the ‘merger of the environmental and civil rights movements’ [9], and importantly, understood justice not solely as the protection of the environment, but of the people whose lives and livelihoods depend on the very environment being threatened [10]. It was in the early 2000s that the concept of climate justice began to be used. In 2001, at COP6, the first Climate Justice Summit was held and resulted in the Environmental Justice and Climate Change Initiative. It noted that the burden of climate change would fall on those most vulnerable to and least responsible for greenhouse gas (GHG) emissions, with greater implications for both the economy, including hardships and unemployment, and health-related impacts on communities due to rising temperatures and pollution [10]. However, it was back in 1992 that climate justice was codified into the global climate regime when the concept of *common but differentiated responsibilities* (CBDR) *and respective capabilities* was set out in Principle 7 of the Rio Declaration: ‘In view of the different contributions to global environmental degradation, States have common but differentiated responsibilities. The developed countries acknowledge the responsibility that they bear in the international pursuit of sustainable development in view of the pressures their societies place on the global environment and of the technologies and financial resources they command’ [11].

The concept of climate justice came to prominence during COP15 in Copenhagen where 100,000 protesters marched on the Bella Conference Centre to voice their anger [12]. Climate justice recognises that countries least responsible for climate change are often those disproportionately affected by its consequences. This structural injustice has its roots in historical global inequalities following centuries of colonisation and exploitation. Today, we still see how the poorest in society, whether they be in Beira, Mozambique or Flint, Michigan, USA, carry a disproportionate share of the impacts of environmental and climate change. In Copenhagen, demonstrators used climate debt to express their anger at these inequalities, highlighting that climate justice is much more complex than a financial transaction, and needs to be seen within the context of centuries of resource exploitation [12].

Climate justice has been explored by scholars, politicians and activists in different ways including how loss and damage (L&D) is framed within negotiations, how justice contributes to norms and the rules that come out of the global climate regime [13], as well as how it is incorporated with technical solutions such as carbon credits [14]. Within the COP processes, CBDR has been tied into climate justice since the inception of the global climate regime in the early 1990s. Originally, the discourse revolved around GHGs and melting ice caps; the assumption being that climate justice was about developed countries investing in low-carbon pathways and providing finance to developing countries as part of a mitigation stimulus package [15]. But carbon and its trade within financial markets are only one aspect of climate justice and may obscure or even undermine actual changes in behavioural patterns and technological changes that need to take place to effectively tackle global warming. For example, the impacts of climate change on land and land tenure have become a human rights and gender issue [16]. More recently, the climate justice discourse has become intertwined with the civil rights movement, as they represent the same thread of domination and exploitation. Through the recent marches, strikes and protests by hundreds of young people across the globe, many have come to see that there is an intergenerational injustice aspect to climate change as young people are now recognised as the means of implementation and creators of opportunities [17].

**Figure 1:**
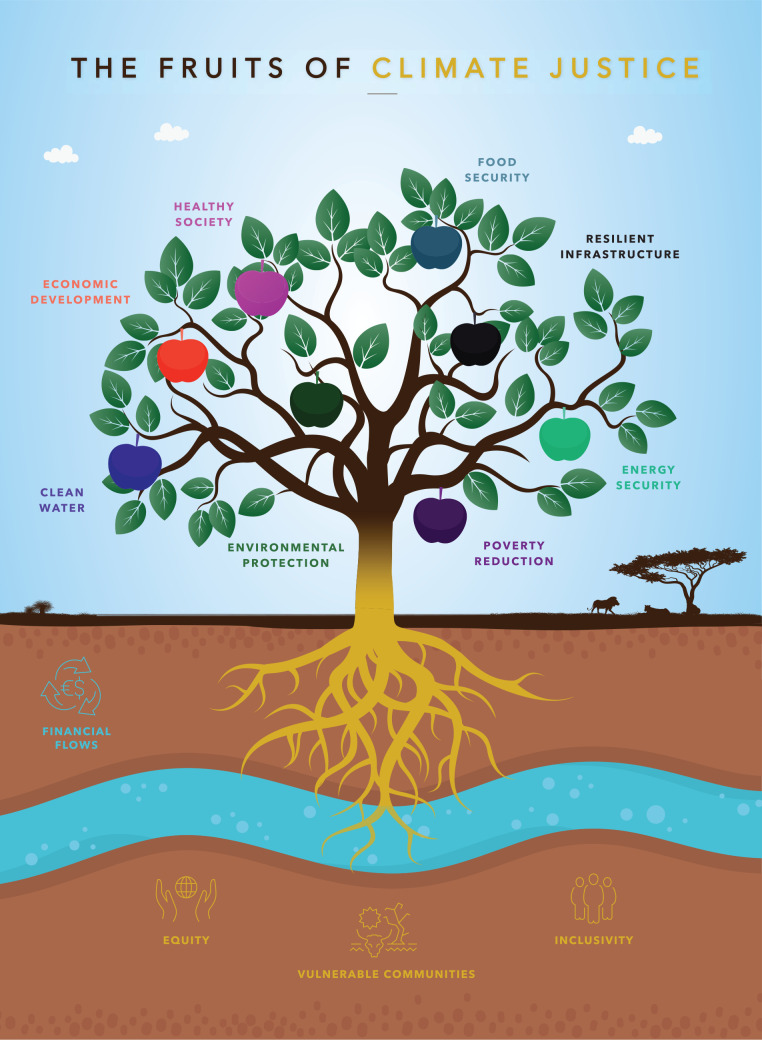
Climate justice is at the root of required climate action.

In April 2022, the IPCC Working Group III released its contribution to the Sixth Assessment Report (AR6). The report focuses on the importance of transitioning away from fossil fuels and looking at the need to re-think energy demands, as well as living standards and requirements for basic human well-being, nutrition, housing, health, education and mobility [18]. The report also contends that climate justice and equity need to be embedded throughout the interventions and approaches to tackle climate change. The IPCC Working Group II also complements this idea where it highlights the role of climate justice in the transition and its use as a marker in identifying effective and successful results (Section 1.4.1) [3]. The reality is that climate justice is more than reducing emissions and being part of the carbon market approach to tackling climate change, it is at the root of the impact (as reflected in Fig. 1). Addressing climate change equitably requires an acceleration of financing and healthy communities. Finance feeds into a greater capacity to protect vulnerable communities that are not always visible but are crucial to climate goals for healthy societies and global poverty reduction. The tree of life concept is not new but rings true across many avenues: without water, roots die and the tree suffers. Climate justice bears good climate fruit and protects all communities. It is also about putting in place effective approaches to decarbonisation, while at the same time allowing for sustainable socio-economic development and a ‘right to exist’ in a just and healthy society that enables people to be food-secure, enjoy clean water, be adequately housed and clothed, as well as having access to health care and education [19].

Here, we look at the role of justice narratives in shaping the recent phenomenon of unity in climate negotiations amongst LDCs, with a focus on the African continent, and how it is used to contest the unequal social and geographical impacts of climate change and what these mean for action on energy, health and the environment. While our focus is on the African continent, within the climate negotiations, countries align themselves with negotiating bodies that best represent their priorities (e.g., unity amongst the LDCs or a common position within the Alliance of Small Island States – AOSIS). Africa is a continent that is particularly susceptible to climate change and extremes in weather patterns and the challenges of human security, water scarcity, food production, natural disasters and sustainable development [20,21]. Existing gaps in access to basic infrastructure services such as water, sanitation, electricity and cooking fuels are likely to be further exacerbated by climate change, impacting vulnerable communities that are already struggling to access resources. African governments have also long asserted that the historical responsibility of carbon emissions resides with the major emitters [22–24]. The Africa Group of Negotiators (AGN) thus claims a moral legitimacy in negotiations [25] as the continent that is most vulnerable and least responsible for anthropogenic climate change. The narrative now includes safeguarding the rights of the most vulnerable people, the fair and equitable sharing of burdens of climate change, securing rights against ‘green land grabbing’ [26], as well as notions of historical responsibility. This green land grabbing essentially points to an unjust development and appropriation of land resources for the environment. The Covid-19 pandemic also provided insight into the consequences of this inequity as many communities lacked access to clean water, inhibiting handwashing as a deterrent to viral transmission and increasing viral exposure from water vendors. Climate change adds a layer of vulnerability to the structural injustice that already plagues societies.

### Supporting vulnerable and marginalised communities

For many African countries and LDCs, vulnerability to climate change is central to understanding their condition/situation, as it informs their narrative [27]. It is a core component of climate justice and the concept of CBDR, which has formed the basis of many developing country’s approaches to the negotiations. Vulnerability can broadly be defined as environments and economic sectors that are particularly susceptible to the changing climate, the socio-political stresses that magnify the issue, and a country’s ability to adapt to these changes [28,29]. Simply put, we can no longer see vulnerability as those exposed to natural disasters but how the most marginalised in society are particularly vulnerable to climatic changes. Indeed, within the climate negotiations, the AGN, as well as AOSIS, have highlighted their vulnerability.

Since the adoption of the Paris Agreement in 2015, all but one African country (Libya) has submitted at least one nationally determined contribution (NDC) to the United Nations Framework Convention on Climate Change (UNFCCC), and many have comprehensive climate action strategies and adaptations plans (see Kenya, Rwanda, Ethiopia, etc.). However, many if not most of these NDCs remain conditional, as without adequate tools (technology, skills, infrastructure, etc.) as well as finance, the ability to implement adaptation projects and policies is limited. This is further exacerbated by how African countries have historically been marginalised in the UNFCCC processes, with more developed countries (MDCs) exerting greater bargaining power to get their way in the negotiations. Additionally, IPCC reports have highlighted that ‘heightened vulnerability is rarely due to a single cause. Rather, it is the product of intersecting socio-economic processes that result in inequalities in status and income, as well as in exposure. Such social processes include, for example, discrimination based on gender, class, ethnicity, age, and (dis)ability’ [30].

Countries across the African continent stand out as particularly vulnerable in many critical sectors and, as such, have special status within the UNFCCC processes. It is widely recognised that Africa is already experiencing temperature rises higher than many other parts of the world [27,28,31]. At COP27 Sharm El Sheik, issues of L&D and climate justice are expected to be central to any advance in negotiations over mitigation and adaptation. In recent years, there has been a stronger focus on the justice aspect of climate negotiations. Where developed countries still frame climate change around extreme weather events, extinctions and mitigation measures, for African countries, as well as many other developing countries, in particular the small island states (four of which are African), climate change is not simply about weather events and national governance [32] but highlights the divide (inequalities) between MDCs and LDCs. The latter being at greater risk of exposure to climate change because of socio-economic factors as well as environmental factors [29,30].

Since the inception of the UNFCCC in 1992, climate-related impacts, such as desertification, failing crops, rising lake temperatures and unpredictable weather patterns have been on the rise across Africa [31]. This has led to widespread food insecurity and economic instability, displacing communities who are in turn coming into conflict over remaining resources [33,34]. For example, the 2011 Somali famine has resulted in an increase in refugees in Djibouti, Ethiopia and Kenya [35], as well as approximately 3 million internally displaced people in Somalia itself [36]. In March 2019, Cyclone Idai devastated Mozambique, Malawi and Zimbabwe, killing more than a thousand people, and leaving 2.6 million in need of humanitarian assistance. The inequalities within communities were on full display, as the hardest hit in the decimated city of Beira were those living in makeshift housing, making them immediately vulnerable to the cyclone and the resulting flooding [37]. The 2014 IPCC assessment also noted the increased surface water temperature in Lakes Kariba, Kivu, Tanganyika, Victoria and Malawi; the increased risk of malaria in the East African Highlands; and reduced meltwaters in the Atlas Mountains leaving less water for the lowland areas of Morocco [38]. These climatic shifts are exacerbated by low levels of development across the continent, in addition to high levels of wealth distribution inequalities. This is often lost in negotiations but forms the foundation upon which climate justice is framed.

### Gender equity

A recent study highlighted that children born within the last two years have been exposed to approximately seven times more extreme climate events compared to those born in the 1960s [39]. Furthermore, women and children are by far the most vulnerable across all climate concerns from impacts on health to economics made worse by increasing poverty. However, while poverty is increasing globally, in regions such as Sub-Saharan Africa, hosting approximately 70% of the world’s impoverished people, the situation is significant [40]. The poverty observed in parts of the region further magnifies the vulnerability of women, who are often less likely to be formally employed and consequently work in sectors that will feel the impact of climate change [41,42]. Studies reveal that those who work in the agricultural sector tend to have limited access to land and resources including tools and fertilisers [42,43]. This imbalance forces more women into a poverty trap, which has knock-on effects on their health and their children [44,45]. Notwithstanding the efforts of countries such as Burkina Faso, which have explicitly identified the vulnerability of women and have curated an action plan with the United Nations Environmental Programme (UNEP) that addresses these concerns as part of the nation’s climate strategy [46], there is still much to be done across the region. Several studies reveal the strong correlation between the shifting climate and access to education, where many are unable to attend due to poor conditions [3,47–51], further exacerbated by reduced access to necessary resources including water (for drinking and sanitation), food and funds. Unfortunately, this leads to a greater concern nationally and regionally as education is fundamental to bridge the inequity gap, reduce poverty and increase the region’s adaptive capacity. Injustice towards vulnerable communities, both active and passive, further destabilises communities and the region. This hinders their capacity to adapt to climate impacts and the associated burdens that accompany it.

### Food and water security

Food and water security in Africa is threatened by increasing temperatures, which amplify evapotranspiration and alter the timing and distribution of precipitation, primarily as rainfall. A key transition is the intensification of rainfall, which leads to fewer but heavier rainfall events, increasing the frequency and intensity of floods as well as the frequency and duration of droughts [52–56]. This amplification of regional hydrological variability has a set of cascading impacts that extend beyond water-supply provision and food production to include hydro-electric power generation and tourism. The increased frequency of climate extremes not only presents challenges to the provision of climate-resilient water-supply infrastructure but also heightens existing inequalities in water-supply provision that was visible during the period leading up to Cape Town’s ‘Day Zero’ in 2018 [57]. With the populations of people living in African drylands (e.g., Niger) projected to double by the middle of this century, risks posed by climate change to water-dependent sectors are magnified and require planning in the face of substantial uncertainty regarding the range of projected climate extremes. Notwithstanding recent evidence of the potential resilience of groundwater resources in African drylands to climate change [58], such pathways to climate-resilient development continue to face severe data constraints and inequities in funding and research leadership to inform adaptation.

Food systems in Africa are threatened by climate change. Crop production is overwhelmingly conducted by smallholder agriculturalists and is rain-fed, with the regional proportion of arable land under irrigation much lower than the global mean. Crop yields and productivity are thus especially vulnerable to changing rainfall regimes (e.g., timing, predictability). This includes, for example, the suppression of the ‘long rains’ from March to May in East Africa [59]. Despite the marginal benefits of increasing atmospheric carbon dioxide (CO_2_) concentrations on plant growth, yield reductions for staple crops are projected for warming beyond 2°C across most of Africa [60–62]. Livestock production and fish harvests, whether freshwater or marine, are affected by heat stress and disruptions in water supplies caused by increased hydrological variability.

Access to water and safeguarding of resources are critical when looking at infrastructural development. In African nations, food and water security is more than a climate issue, it is also seen as a priority issue and speaks to a matter of justice and security. The World Health Organization (WHO) estimates that 2 billion people across the globe lack access to safe drinking water [44]. Sustainable management, monitoring and climate mitigation are paramount to ensure access in the medium and long term. The projected impact of climate change on food security is daunting, particularly when framed against the expected population growth in regions such as Africa to approximately 4 billion by 2100 [61]. Furthermore, as roughly two-thirds of the continent’s population is employed in the agricultural sector, the climate impact goes beyond the environmental and biophysical and is acutely felt in economic terms [3]. The decline in crop yields under global warming [56,63] has led to increased pressure on agriculture to boost food supply [62] and in turn has hindered efforts to stem deforestation in the region. For countries such as Liberia, balancing the scale between social and economic development and sustainable practices to mitigate climate impacts pose a challenge; however, the government is poised to integrate innovative approaches to their agricultural plans to facilitate the growth of palm oil and cocoa with stemming deforestation [63]. A modernisation of the agricultural sector that embeds justice principles in its design, coupled with a shift in diets, is necessary to meet the new demands as sustainably as possible [64–66].

### Environment

The climate change conversation up to now has been dominated by mitigation, the need to reduce global GHG emissions as fast as possible. Despite 30 years of international negotiations GHG emissions continue to rise [67]. Even the Covid-19 pandemic only reduced the annual carbon dioxide emissions to 93% of the 2019 emissions and subsequently emissions have bounced back to pre-pandemic levels despite the pledges at COP26 Glasgow [68]. The unloved twin of the climate change conversation has been adaptation – with clear limitations and negative outlooks seen towards adaptation projects [69]. We see this reflected in the allocation of, and access to, funding, which is predominantly geared towards mitigation projects as well as key climate action conversations largely centred around mitigation as opposed to adaptation. Additionally, some of the policies and projects previously geared towards adaptation have been negatively viewed and considered to perpetuate the conditions that caused climate change [69–72]. At COP27, adaptation is expected to top the agenda. This is because there is a reframing of the climate change challenge into the ‘threat’ or ‘impact’ agenda balanced by the ‘opportunity’ or ‘action’ agenda. The latest IPCC 6th Assessment Report make the impacts of climate change clear and how they scale non-linearly with the average global temperature increase (see Table 1). This familiar narrative, which has been repeated frequently over the last 40 years, is now being balanced by the new opportunities or action narrative – whereby achieving global net zero emissions by 2050 makes everyone safer, healthier and wealthier [67]. The IPCC 6th Assessment Report on *Mitigation of Climate Change* called for rapid and sustained reduction in GHGs and the provision of solutions for all sectors of society, such as a range of essential adaptations to protect people in the most vulnerable countries in the world [18].

**Table 1.** Climate change impact at varying degrees of temperature increases. Table created by Mark Maslin.

°C above pre-industrial levels	Potential impacts of climate change
1.5°C	• Major effects on warm-water coral reef ecosystem. • Significant impacts on vulnerable ecosystems and species (polar regions, wetlands and cloud forests). • Increase in coastal and river flooding. • Increase in extreme weather events. • Increase in the spread of tropical infectious disease. • Increase in heat-related morbidity and mortality.
2°C–3°C	• The Maldives, the Marshall Islands, Tuvalu and many other small island nations have been abandoned. • Major loss of warm-water coral reef ecosystem. • Major changes in the Arctic regions with a substantial loss of Arctic sea ice. • Major increase in extreme weather events and the spread of infectious disease. • Major increase in heat-related morbidity and mortality, especially in the low latitudes. • Significant impacts on vulnerable ecosystems (polar regions, wetlands, cloud forests and mangroves). • Significant increase in coastal and river flooding around the world. • Significant impacts on low latitude fisheries. • Decrease in crop yields and productivity especially in the tropics and sub-tropical regions.
3°C–4°C	• Major impacts on all ecosystems including significant increase in species extinctions. • Loss of all warm-water and many cold-water coral reef ecosystems. • Arctic completely free of sea ice in summer, Arctic temperature increase by 8°C. • Majority of mountain glaciers have disappeared, including all ice on Kilimanjaro (Tanzania). • Major increase in extreme weather events and spread of infectious disease. • Major decreases in agricultural and fishery production and available water resources. • Food and water security become major political and humanitarian issues. • Environmental forced mass migration increases. • Ocean and terrestrial carbon sinks reduce, accelerating climate change.
4°C–5°C	• Catastrophic loss of ecosystems and species all around the world. • Melting of Western Antarctic and Greenland ice sheets accelerate, causing significant rises in global sea level. • Fifth of world population affected by flooding and major coastal cities are abandoned. • Environmental forced mass migration accelerates and there is an increase in conflicts over resources. • In many countries summer temperatures persistently stay above 40°C. • Heatwaves with temperatures as high as 50°C have become common. • Over 3.5 billion people are now water stressed. • Wildfires have created major air pollution events and human health crises. • Global food production plummets, leading to widespread malnutrition and starvation.
5°C–6°C or higher	• Do not go there.

The colours in this table reflect the increase in temperature.

The opportunity/action narrative assumes implicitly the realisation of social justice or environmental justice. Technological solutions can be envisaged which reduce GHG emissions and may even remove GHGs out of the atmosphere, but it leaves billions of people in extreme poverty. Hence, a critical reframing of the climate change conversation is that of climate justice [73]. This is to ensure that there is full inclusion and diversity in decision-making regarding climate and environmental crisis solutions.

We also need to broaden the environmental debate beyond just the headline emergencies – climate change, tropical forest deforestation and plastic pollution. Given the huge impacts humanity has on the planet all aspects of our environment and all ecosystems are under threat [74]. The opportunities narrative must be holistic and include addressing all our environmental impacts and consider the possibilities of repairing our environments and ecosystems. Hence, reforestation, rewilding and recreating environments such as wetlands and tundra need to be openly discussed and assessed in full consultation.

### Infrastructure and development

Inadequate infrastructure heightens the impact of climate change in LDCs and MDCs. Even more so, there is a disparity with the effect in society, where women are targeted to bear the burden. For example, in areas with limited access to water, such as rural Mali and Ethiopia, approximately 40% of young women are held back from schooling to go on long walks in search for water [75]. Similarly, climate emergencies result in women losing jobs [76], women being exposed to more heat stress due to outdoor cooking and searching for water [77] and limitations on their education due to lack of hygiene and sanitation services [78]. Sustainable infrastructure spans beyond mere access to water and instead highlights a limitation to an essential resource that exacerbates sanitation and health concerns. Furthermore, the environmental risks associated with ineffective infrastructure are significant due to informal settlement in flood-prone areas, which exposes communities to waterborne diseases and displacement [77].

Addressing access to water and sanitation is steeped in all 17 Sustainable Development Goals (SDGs) [79] and, as such, addressing climate change holistically can positively shift the impact in LDCs and MDCs [80]. A balanced solution is for increased green infrastructure and sustainable development in these areas [81] to facilitate flood and climate resilient toilets enabled with access to water systems and safe wastewater treatment in schools, health clinics and other key institutions. A report published by the UNFCCC optimistically indicated that approximately USD 7.6–130 billion would be required to adapt new infrastructure to climate change with only around USD 22–371 million of that required for the African continent [82]. It is clear that this will not be enough as there will be a need to provide basic infrastructure to the millions deprived of access to water, electricity, sanitation and hygiene.

Given the infrastructural overhaul and development required on the continent, namely more green infrastructure, it is necessary to re-evaluate how best to foster investment in infrastructure in Africa. At COP27, it is necessary to open a serious discussion on how to enable access to safe drinking water, sanitation and energy infrastructure through adaptation funds to address developmental needs and climate justice simultaneously. Coupled with this, an interconnected arena of green spaces to support ecosystem and human health is necessary [83] as we consider climate action with justice at its centre. With the gaps in infrastructure in Africa, the continent is lagging behind in achieving the SDGs and has been slower to take up new green tech due to lack of engagement with communities impacted by greening measures, as well as the lack of attention to socio-political factors [84]. The continent requires adequate financial support for climate adaptation that is tailored to their needs to retrofit existing infrastructure and develop climate resilient infrastructure. While many MDCs are discussing the implementation of novel technologies in infrastructure, for example, carbon capture for cement or green infrastructure, the majority of infrastructure development, and also emissions, will be in LDCs where these conversations and technologies are far from mainstream.

### Health

Increased temperatures, food and water scarcity, financial instability and imbalanced policies all lead to increased exposure to diseases and other health risks, which are highlighted extensively in the published Lancet Countdown report [2]. The Lancet Countdown is an international, multidisciplinary collaboration reporting on and tracking global indicators of climate change and health. The indicators evaluate climate change health exposures and impacts; climate adaptation; mitigation actions and health co-benefits; economics and finance; and public and political engagement. One of the key conclusions from the report is that health is impacted by variability in other sectors including finance, environment, infrastructure and agriculture. For example, increased temperatures contribute to droughts which affect food security; emissions pollute the atmosphere; insufficient access to finance limits the capacity to support clinics and hospitals; and inadequate infrastructure often leads to a lack of access to water and in some cases exposure to extreme climate scenarios such as floods. Additionally, the health sector makes a significant 5% contribution to global emissions, and there is much scope for making health systems more sustainable, but national adaptation planning is poor. Urban planning and protection of green spaces is the exception rather than the rule as rural to urban migration accelerates in low and middle human development index (HDI) countries [2].

As previously outlined, rising temperatures coupled with a gross impact on food, and water scarcity, have led to a corresponding increase in child hunger, population migration and loss of livestock [2]. The environmental impact of the climate impact has also resulted in rising cases of infectious waterborne diseases with increased warming as studies have shown infectious disease risk has increased for dengue fever, malaria at altitude, and climate conditions favour the mosquito vectors for Zika virus [2]. Warming coastal waters are also increasingly suitable for the transmission of pathogenic *Vibrio cholerae* that cause cholera outbreaks.

Another concern for health is the increased risk of exposure due to mitigating another crisis. For instance, in Sub-Saharan Africa, studies have noted that increased development to meet food security needs may lead to further deforestation and limitations to curb exposure to diseases such as malaria [85,86]. In cases where pesticides and other chemicals are used to strike a balance, there is still the impending impact to the water supply. It becomes a dangerous cycle which at the very core puts poorer communities at the greatest risk. Nevertheless, there is still a brief window of opportunity to ‘shift gears’. Coupled with the pandemic, the Russian invasion of Ukraine and the continued lockdowns in China have severely affected fuel and food prices and reminded countries of the risks of being dependent on a ‘just-in-time’ globalised economy. It is essential that we accelerate renewable energy replacement for fossil fuels, reduce inequalities within countries and fiercely protect ecosystems around the world. If we fail, the future for our children is perilous.

### Energy

Energy is key in the socio-economic development in Africa but is also heavily implicated in the rise of GHG emissions. The production of fossil fuels varies widely across the continent [87] as is the access, or lack thereof, to energy in different regions, which directly correlate to social and economic challenges. While reports like the Lancet Countdown have highlighted the worsening conditions and climate impact on health, they also highlight the limitations due to finance and planning, among others, that inhibit change. There is an understanding that almost all investments to reduce climate change will produce substantial health co-benefits. For example, removing subsidies for fossil fuels, increasing renewable energy, making food production systems more sustainable, local and less wasteful, will produce huge health benefits for populations and cut health care costs. But the Paris Agreement commitments have simply not been met. The adoption of clean energies has been particularly slow in low HDI countries, with only 1.4% of their electricity coming from new renewables in 2020, against 9.5% in very high HDI countries [2,88,89].

With the current geopolitical climate, diversification of energy sources is paramount, especially for developing nations such as those in Africa. While renewables are on the table to move climate action in the right direction, there is growing interest in pushing investment into developing African gas. The rush to exploit gas reserves in Africa is motivated to relieve the dependence of European countries on Russia as well as advance economic development on the continent. That said, the transition to cleaner energy is complicated and expensive. With primary energy companies in these countries in severe debt, coupled with political minefields, poor records and policies, the shift away from coal requires a tailored approach and needs a radical shift in supply chains. At the conclusion of COP26, the European Union (EU) and the United States announced their intention to fill this gap and provide funding to enable a tailored transition from coal. South Africa was one of the beneficiaries, and received USD 8 billion to accelerate its decarbonisation goals [90,91]. The funding is also expected to encourage policy reform of electricity providers as well as enable the protection of vulnerable communities. While such funding opportunities can support developing countries in their transition to clean energy, there is still a question of their effectiveness and sufficiency to address the scale of the challenge faced by Sub-Saharan Africa with 600 million people without access to electricity and 970 million people using polluting cooking fuels [92]. This includes supporting solar, which as a sector is seeing rapid growth, and other renewable energy projects in the region. While there has been an increase in renewable energy use in the region in recent years (up ~2% from 9% since 2019), more work needs to be done around subsidies and policies to support investment in renewables. With the right enabling environment, the International Renewable Energy Agency (IRENA) estimates that Africa could reach 70 GW of solar photovoltaic (PV) capacity by 2030. With the cost of renewable energy declining, there should be a corresponding increase in capacity projects across the continent in the near future to create new jobs and address net zero ambitions.

### Finance

It is difficult to quantify the exact economic impact of different climate futures, but it is undeniable that the impact will be greater in poorer countries [93]. As a result, climate finance, both public and private, is at the root of many of the missteps and lack of necessary action noted in developing countries. It is a concern that has been and continues to be raised in climate negotiations and has not been helped by broken finance promises [94]. Many bemoan the lack of funding for loss and damage in poorer countries; the promises made in Copenhagen to provide USD 100 billion per year for this purpose remains off-track and was a major reason for disagreements at COP26. It is challenging for developing countries to enable effective mitigation and adaptive measures without adequate and timely funding. Such countries often face far greater capital costs for investment due to institutional risk, which may result in a climate investment trap [95]. Africa presently is one of the least insured regions against climate risk (<1%) with an estimated USD 1 billion in projected losses [96].

In recent years, global climate finance stands in stark contrast with the rapid rolling out of over USD 18 trillion for Covid-19 rescue and recovery. This is also evident in the corporate space, where there is a lack of capital injection from corporations towards the climate crisis [97]. The roll-out of carbon taxes has been slow and ineffective [2,98]. According to the Climate Policy Initiative, only 3% of climate finance in 2020 occurred in Sub-Saharan Africa with another 2.5% in North Africa and the Middle East [99]. Estimates suggest it will cost roughly USD 2.8 trillion to achieve Africa’s NDCs by 2030, of which roughly only 10% has been promised by African governments, leaving a shortfall that must be met from either international public funds or from the private sector [100].

**Figure 2:**
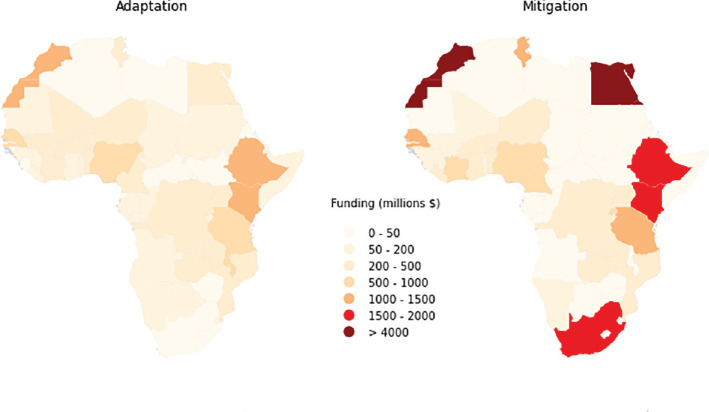
Allocation of climate finance in African Nations [105].

There are many barriers to adequate finance reaching local communities which include shifts in priorities as well as an inability for international bodies to fund local projects directly but through government agencies instead. In fact, it is estimated that only 10% of climate finance makes it to the local level [101,102] while the majority are sitting within groups that lack the capacity and in some cases the will to execute solutions. Furthermore, even with the increase in available financial sources, there are still regions that are unable to access the resource due to inequitable structuring and regulations that are not fit for purpose. The lack of transparency and input from the vulnerable communities exacerbates the situation and prolongs the impacts faced in these developing regions, such as those in Africa. Furthermore, according to the United Nations Economic Commission for Africa (UNECA), many of the countries in this region find themselves having to choose between three dire circumstances: to spend on health care versus climate adaptation versus education. Countries such as Zimbabwe and Cameroon spend 9% of their GDP on climate adaptation, Ethiopia spends 8%, while Sierra Leone, Ghana and Senegal spend around 7% [103,104]. When we consider that the average GDP in Africa is USD 1500 per capita, coupled with ongoing vulnerabilities in the region such as drought in the Sahel region, floods in the Nile Delta, sea level rise impacts in Lagos and tropical cyclones in Mozambique, the tension on the purse is tighter. Ministers in the region have indicated that most of climate funding available is currently earmarked for GHG mitigation but the reality is that adaptation finance is needed and is significantly more expensive than mitigation, and these costs will continue to increase as action is delayed. Figure 2 highlights how funding has been allocated across the continent [105,106]. There is an imbalance in funding allocated towards adaptation in some countries, including Egypt, which is challenging for a region that is already experiencing the effects of climate change. While mitigation efforts are necessary, without funding for adaptation measures in place, countries face greater unnecessary climate risk. Despite avoiding adaptation discussions on the global stage in previous years and hindrances for funding adaptation projects [70], an attempt to somewhat rebalance this divide between mitigation and adaptation was made at COP26 to commit to achieving a doubling of adaptation finance by 2025.

If MDCs cannot agree on increased public funding, then the least they can do is enact processes to help shape the way private funding flows through the introduction of various policy options such as targeted lending, weather indexed insurance and/or national climate funds to name but a few [105]. Also, there are other adaptation solutions currently being tested in parts of the continent including the Climate and Ocean Risk Vulnerability Index (CORVI) tool [107], which can be used to collect data and conduct surveys. The information generated have been used to tailor Kenya and Tanzania’s national policies. Nevertheless, these options have their pros and cons, but their effectiveness is rooted in proper enforcement and integrity. As we approach COP27 with the promises from Glasgow, it is evident that a fair and equitable coordination of climate finance will play a key role. However, a prerequisite to a successful approach is one that considers flexibility, in that as climate impacts create new challenges, the policies and frameworks around climate finance need to be dynamic enough to adjust. This flexibility facilitates effective support where and when needed [104].

## The way forward: a justified shift for climate action

Moving forward with climate action requires a radical shift from the normative discourse. There is a fundamental problem with the international institutions addressing the climate and environmental crisis. While they have seen some successes in the past three decades – for example, the (not so) simple fact that 196 member states were able to come to consensus on adopting the Paris Climate Change Agreement should not be underestimated – in their current configuration, they have still not been able to adequately adapt to the fast-paced changes in both the environment as well as the socio-economic and political landscape of today’s world. Climate justice is not a new concept in the global climate change regime, but the inequalities that divide the negotiations continue. For example, the World Bank was formed in 1944, the United Nations (UN) and the International Monetary Fund (IMF) were formed in 1945, and the General Agreement on Tariffs and Trade (GATT) was signed in 1947 which was replaced by the World Trade Organization (WTO) in 1995. At the bourgeoning of these organisations a vision was established to meet the economic needs of regions and countries based on past conditions that we are now beyond. We need our international institutions to represent everyone, not only in today’s world, but far into the future to ensure fair and equitable governance. There is a need for a 21st century set of Bretton Woods institutions to improve or change international governance, making them more reflective and efficient to address the cascade of global challenges facing the world today [108]. This should include redesigning the World Bank and IMF, so they focus on developing the green sustainable economies and alleviating poverty. Restructuring how national and international organisations address all avenues of climate change is crucial in this fast-changing world.

Reliance on the global fossil fuel industry, as it stands today, will not keep global temperatures to below 2°C, let alone the 1.5°C as set out in Paris. The whole energy industry needs to be rethought and supply chains need to be reconfigured. The lobbying by companies in fossil fuel-rich countries and those accepting their funding needs to be called out and redirected to alternative energy sources. The Glasgow Climate Pact called for a phasedown of coal and the phase out of inefficient fossil fuels subsidies [109]. The WTO encourages trade and consumption, making reductions in carbon emissions and meaningful local, national and international environmental protections and regulations challenging to implement. While the WTO has its own pitfalls and divides between countries, where once again, the more powerful countries can dominate the smaller economies, the strength of the WTO is in its enforcement mechanisms to get states to adhere to their commitments. The current global climate regime relies on voluntary actions by states. The creation of a World Sustainability Organisation (WSO) that would have the ‘teeth’ to enforce states to live up to their commitments made in the NDCs is required. There is a need to recognise that free trade is not always in the best interests of developing countries. Since 1960, the income gap between MDCs and LDCs has tripled in size. Since 1980, over USD 16.3 trillion has been transferred from the least developed regions to the developed regions [110]. Of this over USD 4.2 trillion was in interest payments, direct cash transfers to big banks in New York and London [111].

The language around climate change needs certainty. While scientific evidence points to the actual impacts of climate change, ‘likely’ impacts leave space for ambiguity and inaction from policy makers operating with a defined mindset. We also see uncertainty in the language presented from COP and other international negotiations. It is clear, particularly when the global climate regime keeps calling climate change and international crisis, but governments are not acting like we are in an international crisis, this lightens the pressure needed to push stakeholders to make timely decisions on climate action. Enabling the above requires us to look at the intergovernmental and global agencies and assess their structures for the future. For example, the UNEP has secondary status within the UN system. It does not have the same status as trade, health, labour or even maritime affairs, intellectual property or tourism. UNEP’s budget is small, significantly less than that of other UN bodies such as the WHO and a tenth of the World Food Programme despite being central to both organisation’s mandates [112,113]. UNEP should be upgraded to a full UN World Environment Organisation (WEO). The WEO would require a budget that is at least the size of the WHO. The WEO should incorporate the SDGs, the Convention on Biological Diversity, the Convention to Combat Desertification, as well as the climate change negotiations to ensure they are mutually reinforcing and not in opposition. This would allow it to function as an umbrella organisation, tracking the over 500 multilateral and bilateral environmental agreements that already exist. Having a WEO or a WSO with more teeth and the ability to enforce action on both decarbonisation and adaptation projects is essential. For instance, during Rio+20 (2012), Kenya outlined the importance of an international environmental organisation. As Nairobi already hosts a UNEP, they proposed to transform this programme into an organisation with an authoritative voice. While it was rejected by member states, there are African countries that see a WEO as an equitable way forward that would grant a new specialised UN organisation the agency to go beyond what the UNFCCC or UNEP can currently achieve. While the argument that yet another UN organisation in the already saturated archaic global system would further muddy the water, we argue that as global coordination and monitoring body, it would have the ability to effectively streamline all existing agreements, conventions and treaties, while at the same time giving climate justice a central role in allocating resources for adaptation programmes.

At the more localised level, key funding to subgovernment and local initiatives, such as the African-led Great Green Wall (GGW), will be important for the hands-on action on climate change and need to be supported to facilitate adaptation initiatives. As one of the earliest collaborative initiatives in Africa, the GGW initiative represents targeted resilience across African nations with an aim to combat land degradation and desertification across the Sahel region, particularly as the region faces land degradation due to human impact and climate change [114,115]. While the initiative is politically supported and led by the countries in the Sahel region, it still faces significant environmental, economic and social challenges in implementation. This presents an opportunity for transnational unity within the African Union, one that builds on and improves knowledge that is interdisciplinary and decentralised for regional benefit. The goals under the initiative set the region on achieving 15 out of the 17 SDGs as well as meeting targets outlined in the Africa Agenda. An agenda that highlights targeting solutions that are tailored for the region but beyond this, sub-governmental and local groups have insight on solutions that are key for their communities, their countries, their regions and the continent.

Since the adoption of the Paris Agreement, African countries have submitted at least one round of NDCs and are in the process of tackling global climate change. As the most vulnerable but least responsible for the changing climate, they have demanded climate justice. These local and national actions on the continent contribute to global climate action. It is crucial to understand that countries across the continent do not experience climate impacts and inequity homogenously. Africa’s emissions also vary widely, with 87% of the emissions coming from just 10 countries in 2017 [88]. Their priorities and energy needs continue to vary and grow across the continent. Saying that, within the UNFCCC process, they negotiate as a continent and come up with positions (Africa Common Position) that are intended to represent the needs and priorities of the continent as a whole [28]. It is important for the aforementioned financial organisations, among others, to recognise the limitations that their current framework has for supporting regions such as Africa, and that a shift to focus on localisation is necessary. This means enabling key infrastructure and skills growth in the region, supporting development in tandem with emission reduction across different sectors as well as supporting key initiatives from subgroups that work in the best interest of communities and not necessarily based on what a lending organisation deems fit.

**Figure 3:**
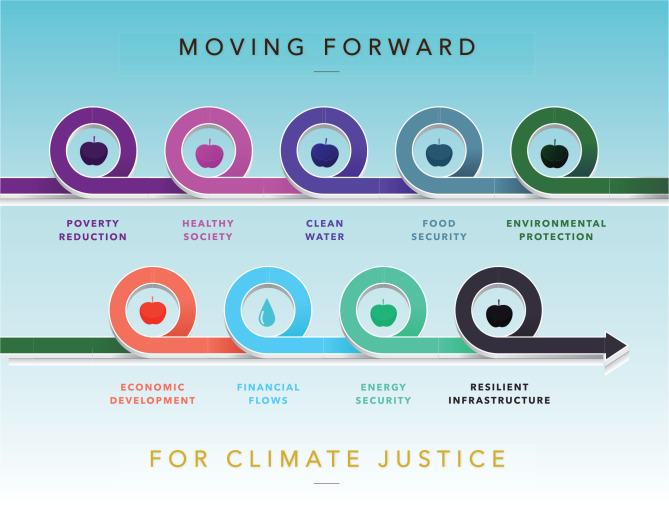
Connectivity in The Way Forward.

While a complete restructuring of international and intergovernmental agencies is necessary, we recognise that the probability of execution is low. Nevertheless, it can serve as a reminder that the current infrastructure and systems are simply not working. Heading to COP27 in Africa offers the region an opportunity to highlight the concerns and needs of the continent, putting its interest at the forefront of climate discussions. Finance has been central to the Africa Group and remains key to both the impediment and answer to climate justice. Without funding, adaptation projects will not be implemented, and emissions will not be reduced. That said, these COPs and international summits are the forum in which rules and norms around climate action are negotiated, but the actual action takes place in the months and years following a conference. The region has been and should continue to be steadfast in their interest in climate action but also would benefit from greater collaborative partnerships between researchers and those in the field (e.g., agriculture and fisheries sectors) to create and inform adaptation practices, cross-border collaboration for resources (water resources in particular) and, more importantly, a reformed appreciation for the impact of gender inequity. If climate justice is at the heart of the negotiations, it will be a constant reminder of the true scope of the inequality that exists, but also, as shown in Fig. 3, may provide a pathway forward for actual adequate action on climate change at the global level.

## Conclusion

The impact of climate change is disproportionately greater in developing countries in all sectors, from health to infrastructure to the economy. The risks are great. We know this. And yet, as of now, the strategies to tackle the risks of climate change have been far from successful. The slow approach often leaves vulnerable communities exposed for longer periods of time and at greater risk. Acknowledging climate justice at the core of climate action and solutions establishes a baseline from which we need to operate to have the greatest positive impact on vulnerable communities. Most effectively, it will inform how we approach climate finance both at the local and the national level with a stronger emphasis on climate adaptation to address multiple vulnerabilities. In addition to a restructuring of international bodies, we propose that two key solutions to enable greatest impact are supporting developing regions with adequate and accessible finance and enabling their adaptation plans with tailored solutions that are effective for their respective capabilities and conditions. Otherwise, it becomes a cycle of small, siloed projects with negligible impact, even less so for marginalised and vulnerable communities. Integrated climate policies for finance and other sectors for the future must be able to effectively adapt to changing circumstances locally, and regionally.

## Data Availability

Data sharing not applicable to this article as no datasets were generated or analysed during the current study.

## References

[1] Coffel ED, Horton RM, de Sherbinin A (2018). Temperature and humidity based projections of a rapid rise in global heat stress exposure during the 21st century. Environ Res Lett.

[2] Romanello M, McGushin A, di Napoli C, Drummond P, Hughes N, Jamart L (2021). The 2021 report of the Lancet Countdown on health and climate change: code red for a healthy future. Lancet.

[3] Trisos CH, Adelekan IO, Totin E, Ayanlade A, Efitre J, Gemeda A (2021). Africa. Climate change 2021: impacts, adaptation and vulnerability. Contribution of Working Group II to the Sixth Assessment Report of the Intergovernmental Panel on Climate Change. [online].

[4] IPBES (2019). Summary for policymakers of the global assessment report on biodiversity and ecosystem services of the Intergovernmental Science-Policy Platform on Biodiversity and Ecosystem Services. Popul Dev Rev.

[5] IPCC (2022). IPCC, 2022: summary for policy makers. In: Climate change 2022: impacts, adaptation and vulnerability.

[6] World Bank (2016). World Bank Group Climate Action Plan 2021–2025. The World Bank Group A to Z.

[7] German Development Institute (2022). Global Solutions Initiative. Think 7 (2022) Communiqué G7. [online].

[8] Pasquini L, van Aardenne L, Godsmark CN, Lee J, Jack C (2020). Emerging climate change-related public health challenges in Africa: a case study of the heat-health vulnerability of informal settlement residents in Dar es Salaam, Tanzania. Sci Total Environ.

[9] Bryant B, Mohai P (2019). Race and the incidence of environmental hazards: a time for discourse.

[10] Schlosberg D, Collins LB (2014). From environmental to climate justice: climate change and the discourse of environmental justice. Wiley Interdiscip Rev Clim Change.

[11] United Nations (1992). Report of the United Nations Conference on Environment and Development, Rio de Janeiro, 3–14 June 1992. [online].

[12] Chatterton P, Featherstone D, Routledge P (2013). Articulating climate justice in Copenhagen: antagonism, the commons, and solidarity. Antipode.

[13] Calliari E, Serdeczny O, Vanhala L (2020). Making sense of the politics in the climate change loss & damage debate. Glob Environ Change.

[14] Pettit J (2004). Climate justice: a new social movement for atmospheric rights. IDS Bull.

[15] Lohmann L (2008). Carbon trading, climate justice and the production of ignorance: ten examples. Development.

[16] Ali DA, Deininger K, Goldstein M (2014). Environmental and gender impacts of land tenure regularization in Africa: pilot evidence from Rwanda. J Dev Econ.

[17] Calmon D, Jacovetti C, Koné M (2021). Agrarian climate justice as a progressive alternative to climate security: Mali at the intersection of natural resource conflicts. Third World Q.

[18] IPCC Working Group II (2022). IPCC AR6 Working Group II: summary for policymakers: climate change 2022, impacts, adaptation and vulnerability. Implementing a US Carbon Tax: challenges and debates.

[19] Sen A (2021). The political economy reader: contending perspectives and contemporary debates.

[20] Stabinsky D (2011). Climate change impacts on agriculture in Africa and the UNFCCC negotiations: policy implications of recent scientific findings. Working Paper.

[21] McIlvain Moran A, Mulugetta Y, Raleigh C (2014). Climate change & security in Africa: clear risks, nuanced impacts.

[22] Surie von Czechowski A (2020). CDP Africa Report: benchmarking progress towards climate safe cities, states, and regions.

[23] Canadell JG, Raupach MR, Houghton RA (2009). Anthropogenic CO_2_ emissions in Africa. Biogeosciences.

[24] Kassam A (2009). Climate change in Africa. By C. Toulmin.

[25] Chin-Yee S (2016). Africa and the Paris climate change agreement. Afr Aff (Lond).

[26] Franco JC, Borras SM (2019). Grey areas in green grabbing: subtle and indirect interconnections between climate change politics and land grabs and their implications for research. Land Use Policy.

[27] Chin-Yee S, Nielsen TD, Blaxekjær LØ (2020). One voice, one Africa: the African group of negotiators. In: Coalitions in the climate change negotiations.

[28] Adger WN, Huq S, Brown K, Declan C, Mike H (2003). Adaptation to climate change in the developing world. Prog Dev Stud.

[29] Methmann C, Oels A (2013). Critical environmental politics.

[30] IPCC (2014). Summary for policymakers. In: Climate change 2014: mitigation of climate change. Contribution of Working Group III to the Fifth Assessment Report of the Intergovernmental Panel on Climate Change.

[31] Collier P, Conway G, Venables T (2008). Climate change and Africa. Oxf Rev Econ Policy.

[32] Varley A, Varley A (1994). Disasters, development and environment.

[33] Black R, Adger WN, Arnell NW, Dercon S, Geddes A, Thomas D (2011). The effect of environmental change on human migration. Glob Environ Change.

[34] Brown O, Hammill A, McLeman R (2007). Climate change as the ‘new’ security threat: implications for Africa. Int Aff.

[35] Ferris E (2012). Internal displacement in Africa: an overview of trends and opportunities. In: Brookings – LSE project on internal displacement – conference.

[36] Hanmer L, Rubiano-Matulevich E (2022). Want to keep internally displaced people in Somalia out of poverty? Increase women’s economic opportunities. [online].

[37] Oxfam International (2020). After the storm: one year on from Cyclone Idai. [online].

[38] Niang I, Ruppel OC, Abdrabo MA, Essel C, Lennard C, Padgham J, Barros VR, Field CB, Dokken DJ, Mastrandrea MD, Mach KJ (2014). Climate change 2014: impacts, adaptation, and vulnerability. part B: regional aspects. contribution of working group II to the fifth assessment. intergovernmental panel on climate change.

[39] Thiery W, Lange S, Rogelj J, Schleussner CF, Gudmundsson L, Seneviratne SI (2021). Intergenerational inequities in exposure to climate extremes. Science.

[40] Dorward A, Kydd J, Morrison J, Urey I (2004). A policy agenda for pro-poor agricultural growth. World Dev.

[41] Flatø M, Muttarak R, Pelser A (2017). Women, weather, and woes: the triangular dynamics of female-headed households, economic vulnerability, and climate variability in South Africa. World Dev.

[42] Davidson D (2016). Gaps in agricultural climate adaptation research. Nat Clim Chang.

[43] Perez C, Jones EM, Kristjanson P, Cramer L, Thornton PK, Förch W (2015). How resilient are farming households and communities to a changing climate in Africa? A gender-based perspective. Glob Environ Change.

[44] Sesmero J, Ricker-Gilbert J, Cook A (2018). How do African farm households respond to changes in current and past weather patterns? A structural panel data analysis from Malawi. Am J Agric Econ.

[45] Dercon S, Christiaensen L (2011). Consumption risk, technology adoption and poverty traps: evidence from Ethiopia. J Dev Econ.

[46] United Nations Environment Programme (2019). Capacity building for Burkina Faso’s transparency system for climate change mitigation and adaptation.

[47] Marchetta F, Sahn DE, Tiberti L (2019). The role of weather on schooling and work of young adults in Madagascar. Am J Agric Econ.

[48] Alderman H (2006). Long term consequences of early childhood malnutrition. Oxf Econ Pap.

[49] Abiona O (2017). Adverse effects of early life extreme precipitation shocks on short-term health and adulthood welfare outcomes. Rev Dev Econ.

[50] Björkman-Nyqvist M (2013). Income shocks and gender gaps in education: evidence from Uganda. J Dev Econ.

[51] Randell H, Gray C (2019). Climate change and educational attainment in the global tropics. Proc Natl Acad Sci USA.

[52] Masih I, Maskey S, Mussá FEF, Trambauer P (2014). A review of droughts on the African continent: a geospatial and long-term perspective. Hydrol Earth Syst Sci.

[53] Gownaris NJ, Rountos KJ, Kaufman L, Kolding J, Lwiza KMM, Pikitch EK (2018). Water level fluctuations and the ecosystem functioning of lakes. J Great Lakes Res.

[54] Ogutu-Ohwayo R, Natugonza V, Musinguzi L, Olokotum M, Naigaga S (2016). Implications of climate variability and change for African lake ecosystems, fisheries productivity, and livelihoods. J Great Lakes Res.

[55] Natugonza V, Ogutu-Ohwayo R, Musinguzi L, Olokotum M, Naigaga S, Kitabona J (2016). Implications of climate warming for hydrology and water balance of small shallow lakes: a case of Wamala and Kawi, Uganda. Aquat Ecosyst Health Manag.

[56] Descroix L, Guichard F, Grippa M, Lambert L, Panthou G, Mahé G (2018). Evolution of surface hydrology in the Sahelo-Sudanian strip: an updated review. Water (Basel).

[57] Maxmen A (2018). As Cape Town water crisis deepens, scientists prepare for ‘Day Zero’. Nature.

[58] Cuthbert MO, Taylor RG, Favreau G, Todd MC, Shamsudduha M, Villholth KG (2019). Observed controls on resilience of groundwater to climate variability in sub-Saharan Africa. Nature.

[59] Muller A, Olesen J, Smith L, Davis J, Dyrtrtová K, Gattinger A (2012). Reducing global warming and adapting to climate change: the potential of organic agriculture. Working Papers in Economics 526.

[60] Rigaud KK, Sherbinin A de, Jones B, Bergmann J, Clement V, Ober K (2018). Groundswell – preparing for Internal Climate Migration.

[61] Gerland P, Raftery AE, Ševčíková H, Li N, Gu D, Spoorenberg T (2014). World population stabilization unlikely this century. Science.

[62] Jayne TS, Chamberlin J, Headey DD (2014). Land pressures, the evolution of farming systems, and development strategies in Africa: a synthesis. Food Policy.

[63] Niesten ET (2020). Targeted Scenario Analysis (TSA): sustainable palm oil concessions in Liberia.

[64] Foley JA, Ramankutty N, Brauman KA, Cassidy ES, Gerber JS, Johnston M (2011). Solutions for a cultivated planet. Nature.

[65] Mendelsohn R, Dinar A (1999). Climate change, agriculture, and developing countries: does adaptation matter?. World Bank Res Obs.

[66] Howden SM, Soussana JF, Tubiello FN, Chhetri N, Dunlop M, Meinke H (2007). Adapting agriculture to climate change. Proc Natl Acad Sci U S A.

[67] Maslin M (2021). Bill Gates, how to avoid a climate disaster: solutions we have and the breakthroughs we need. Society.

[68] Friedlingstein P, O’Sullivan M, Jones MW, Andrew RM, Hauck J, Olsen A (2020). Global carbon budget 2020. Earth Syst Sci Data.

[69] Eriksen S, Schipper ELF, Scoville-Simonds M, Vincent K, Adam HN, Brooks N (2021). Adaptation interventions and their effect on vulnerability in developing countries: help, hindrance or irrelevance?. World Dev.

[70] Atteridge A, Remling E (2018). Is adaptation reducing vulnerability or redistributing it?. Wiley Interdiscip Rev Clim Change.

[71] Magnan AK, Schipper ELF, Burkett M, Bharwani S, Burton I, Eriksen S (2016). Addressing the risk of maladaptation to climate change. Wiley Interdiscip Rev Clim Change.

[72] Work C, Rong V, Song D, Scheidel A (2019). Maladaptation and development as usual? Investigating climate change mitigation and adaptation projects in Cambodia. Clim Policy.

[73] Cripps E (2022). What climate justice means and why we should care.

[74] Lewis SL, Maslin MA (2018). Welcome to the Anthropocene. IPPR Progress Rev.

[75] Becerra S, Saqalli M, Gangneron F, Dia AH (2016). Everyday vulnerabilities and ‘social dispositions’ in the Malian Sahel, an indication for evaluating future adaptability to water crises?. Reg Environ Change.

[76] Aguilar Revelo L Training manual on gender and climate change, IUCN: International Union for Conservation of Nature.

[77] Reckien D, Creutzig F, Fernandez B, Lwasa S, Tovar-Restrepo M, Mcevoy D (2017). Climate change, equity and the Sustainable Development Goals: an urban perspective. Environ Urban.

[78] UNICEF The Ripple effect: climate change and children’s access to water and sanitation.

[79] Fuso Nerini F, Sovacool B, Hughes N, Cozzi L, Cosgrave E, Howells M (2019). Connecting climate action with other sustainable development goals. Nat Sustain.

[80] Parikh P, Diep L, Hofmann P, Tomei J, Campos LC, Teh TH (2021). Synergies and trade-offs between sanitation and the sustainable development goals. UCL Open Environ.

[81] Tozer L, Hörschelmann K, Anguelovski I, Bulkeley H, Lazova Y (2020). Whose city? Whose nature? Towards inclusive nature-based solution governance. Cities.

[82] Parry M, Arnell N, Berry P, Dodman D, Fankhauser S, Hope C (2009). Assessing the costs of adaptation to climate change: a review of the UNFCCC and other recent estimates. Int Inst Environ Dev.

[83] Benedict MA, McMahon ET (2006). Green infrastructure: linking landscapes and communities.

[84] Matthews T, Lo AY, Byrne JA (2015). Reconceptualizing green infrastructure for climate change adaptation: barriers to adoption and drivers for uptake by spatial planners. Landsc Urban Plan.

[85] Chaves LSM, Fry J, Malik A, Geschke A, Sallum MAM, Lenzen M (2020). Global consumption and international trade in deforestation-associated commodities could influence malaria risk. Nat Commun.

[86] Ordway EM, Asner GP, Lambin EF (2017). Deforestation risk due to commodity crop expansion in sub-Saharan Africa. Environ Res Lett.

[87] Ayompe LM, Davis SJ, Egoh BN (2020). Trends and drivers of African fossil fuel CO_2_ emissions 1990-2017. Environ Res Lett.

[88] Sasmaz MU, Sakar E, Yayla YE, Akkucuk U (2020). The relationship between renewable energy and human development in OECD countries: a panel data analysis. Sustainability (Switzerland).

[89] Azam A, Rafiq M, Shafique M, Yuan J, Salem S (2021). Human Development Index, ICT, and renewable energy-growth nexus for sustainable development: a novel PVAR analysis. Front Energy Res.

[90] Foreign Commonwealth & Development Office (FDCO), The Rt Hon Alok Sharma MP (2022). Alok Sharma joins the South African Government for a ministerial roundtable. [online].

[91] South African Government (2021). Presidency on international partnership to support a just transition to a low carbon economy and a climate resilient society. [online].

[92] International Energy Agency (IEA) (2019). Africa energy outlook 2019: world energy outlook special report.

[93] Tol RSJ (2018). The economic impacts of climate change. Rev Environ Econ Policy.

[94] Timperley J (2021). The broken $100-billion promise of climate finance – and how to fix it. Nature.

[95] The World Bank (2022). Carbon pricing dashboard. [online].

[96] Ameli N, Dessens O, Winning M, Cronin J, Chenet H, Drummond P (2021). Higher cost of finance exacerbates a climate investment trap in developing economies. Nat Commun.

[97] Clark R, Reed J, Sunderland T (2018). Bridging funding gaps for climate and sustainable development: pitfalls, progress and potential of private finance. Land Use Policy.

[98] Swiss Re Group (2022). Natural catastrophes: closing the protection gap together. [online].

[99] Buchner B, Naran B, Fernandez P, Padmanabhi R, Rosane P, Solomon M (2021). Global landscape of climate finance 2021. [online].

[100] Guzmán S, Dobrovich G, Balm A, Meattle C (2022). The state of climate finance in Africa: climate finance needs of African countries. [online].

[101] International Institute for Environment and Development (IIED) Climate finance not reaching the local level. [online]. Mobilising money to where it matters.

[102] Soanes M, Rai N, Steele P, Shakya C, Macgregor J (2017). Delivering real change getting international climate finance to the local level. [online].

[103] Economic Commission for Africa (2017). Africa spending more than its fair share for climate adaptation, a new study reveals. [online].

[104] Hassan GM (2021). Opinion: Africa is living the climate crisis now – COP26 must deliver. [online]. unclimatesummit.org.

[105] Bhandary RR, Gallagher KS, Zhang F (2021). Climate finance policy in practice: a review of the evidence. Clim Policy.

[106] Savvidou G, Atteridge A, Omari-Motsumi K, Trisos CH (2021). Quantifying international public finance for climate change adaptation in Africa. Clim Policy.

[107] Stimson Climate and Ocean Risk Vulnerability Index (CORVI) Tool. [online].

[108] Capie F, Caprio G, Bacchetta P, Barth JR, Hoshi T, Lane PR, Mayes DG (2013). Handbook of Safeguarding Global Financial Stability.

[109] Lewis SL, Maslin MA (2020). The human planet: how we created the Anthropocene. Global Environ.

[110] Hickel J (2017). Is global inequality getting better or worse? A critique of the World Bank’s convergence narrative. Third World Q.

[111] Hickel J (2017). The Guardian.

[112] McArthur JW, Rasmussen K (2018). Who actually funds the UN and other multilaterals? Brookings. [online].

[113] United Nations Environment Programme (UNEP) (2021). Funding and partnerships. [online].

[114] Elagib NA, Khalifa M, Babker Z, Musa AA, Fink AH (2021). Demarcating the rainfed unproductive zones in the African Sahel and Great Green Wall regions. Land Degrad Dev.

[115] Goffner D, Sinare H, Gordon LJ (2019). The Great Green Wall for the Sahara and the Sahel Initiative as an opportunity to enhance resilience in Sahelian landscapes and livelihoods. Reg Environ Change.

